# Gastrointestinal perforation associated with bevacizumab in metastatic colorectal cancer

**DOI:** 10.1002/cnr2.1952

**Published:** 2024-01-22

**Authors:** Kunpeng Fang, Jie Wang, Jianyong Yuan, Chengjun Sui, Jiajun Zhi, Yong Xia, Minmin Sun

**Affiliations:** ^1^ Department of Special Treatment I Third Affiliated Hospital of Naval Medical University (Eastern Hepatobiliary Surgery Hospital) Shanghai China; ^2^ Department of Hepatic Surgery II Third Affiliated Hospital of Naval Medical University (Eastern Hepatobiliary Surgery Hospital) Shanghai China; ^3^ Hepatobiliary Pancreatic Surgery, Yueyang Hospital of Integrated Traditional Chinese and Western Medicine, Shanghai University of Traditional Chinese Medicine Shanghai China; ^4^ Department of Colorectal and Anal Surgery Xin Hua Hospital Affiliated to Shanghai Jiao Tong University School of Medicine Shanghai China; ^5^ Department of Hepatic Surgery IV Third Affiliated Hospital of Naval Medical University (Eastern Hepatobiliary Surgery Hospital) Shanghai China; ^6^ Department of Hepatic Surgery I Third Affiliated Hospital of Naval Medical University (Eastern Hepatobiliary Surgery Hospital) Shanghai China

**Keywords:** adverse reactions, bevacizumab, colorectal cancer, gastrointestinal perforation

## Abstract

**Objective:**

To investigate the risk factors for gastrointestinal perforation in metastatic colorectal cancer patients receiving bevacizumab.

**Methods:**

We retrospectively reviewed 217 patients with metastatic colorectal cancer receiving bevacizumab to investigate the risk factors for gastrointestinal perforation. Three patients occurred intestinal perforation after receiving bevacizumab. We analyzed the clinical characteristics of three patients with intestinal perforation.

**Results:**

All patients receiving bevacizumab. Three of 217 patients occurred intestinal perforation after receiving bevacizumab. Patient no. 1 was 70 years old, female, having history of intestinal obstruction. The patient occurred intestinal perforation and ultimately died after receiving bevacizumab. Patient no. 2 was 59 years old, female, having history of intestinal obstruction. The patient occurred intestinal perforation after receiving bevacizumab, and recovered smoothly after symptomatic treatment. Patient no. 3 was 60 years old, female, having history of intestinal obstruction. The patient occurred intestinal perforation and ultimately died after receiving bevacizumab.

**Conclusions:**

Patients with advanced colorectal cancer receiving bevacizumab are at risk of gastrointestinal perforation. The patient's age, gender and history of bowel obstruction may be associated with gastrointestinal perforation.

## 1 INTRODUCTION

Colorectal cancer is a common gastrointestinal malignancy, accounting for the third most common malignancy of new cases and the second highest number of tumor‐related deaths globally.^1^ The prognosis of colorectal cancer varies greatly due to tumor stage. Studies reported that the 5‐year overall survival (OS) was more than 60% after surgery for early and middle stage colorectal cancer patients.^2^, ^3^ However, the OS was less than 1 year for patients with advanced colorectal cancer.^4^, ^5^ The treatments of colorectal cancer include surgery, chemotherapy, targeted therapy, radiotherapy and so on. Surgery is still the most effective treatment.^6^ However, for patients with advanced colorectal cancer who are unable to accept surgery, chemotherapy or targeted therapy is the better treatments.^7^ Bevacizumab can be used to treat patients with advanced colorectal cancer. Its mechanism of action is to inhibit tumor angiogenesis by specifically binding and blocking vascular endothelial growth factor (VEGF).^8^ It has been clinically proved that bevacizumab is effective for some patients with advanced colorectal cancer and can improve their survival.^9^, ^10^ However, some patients may have some adverse reactions after receiving bevacizumab. Among them, gastrointestinal perforation is the most serious adverse reactions, which can lead to serious consequences and even death. The incidence of gastrointestinal perforation after receiving bevacizumab is reported to be 0%–2%.^11^ Due to the low incidence rate, there is no clear risk factor for gastrointestinal perforation after receiving bevacizumab. This study reported 217 patients receiving bevacizumab in Shanghai Xinhua Hospital and Eastern Hepatobiliary Surgery Hospital from 2015 to 2017, of which 3 patients occurred gastrointestinal perforation. By analyzing the characteristics of these patients with gastrointestinal perforation after receiving bevacizumab, we aimed to explore potential risk factors for gastrointestinal perforation. This provides a basis for the clinical prevention and treatment of such patients in clinical.

## 2 METHODS

### 2.1 Patients

We retrospectively reviewed 217 patients with metastatic colorectal cancer in Shanghai Xinhua Hospital and Eastern Hepatobiliary Surgery Hospital from 2015 to 2017. All patients received mFOLFOX6, FOLFOX4 or FOLFIRI chemotherapy and bevacizumab. Of these patients, 3 patients occurred intestinal perforation after receiving bevacizumab.

### 2.2 Treatment

mFOLFOX6: 2 h infusion of leucovorin 400 mg/m^2^ on day 1 followed by a fluorouracil bolus 400 mg/m^2^ and 46 h infusion 2400 mg/m^2^ every 46 h every 2 weeks, with oxaliplatin 100 mg/m^2^ as a 2 h infusion on day 1.^12^

FOLFOX4: 2 h infusion of oxaliplatin 85 mg/m^2^ on day 1, and leucovorin 200 mg/m^2^ followed by a fluorouracil bolus 400 mg/m^2^ and 22 h infusion 600 mg/m^2^ on day 1 and day 2.^13^

FOLFIRI: 2 h infusion of leucovorin 400 mg/m^2^ on day 1 followed by a fluorouracil bolus 400 mg/m^2^ and 46 h infusion 2400 mg/m^2^ every 46 h every 2 weeks, with irinotecan 180 mg/m^2^ as a 2 h infusion on day 1.^12^

### 2.3 Statistical analysis

The clinical data of patients were recorded using EpiDate 3.1. All data was analyzed using SPSS version 26 (SPSS Inc., Chicago, IL). The measurement data was expressed as median (IQR). The numerical data was expressed as number (percentage).

## 3 RESULTS

Table 1 shows the basic information of the 217 patients. Among these patients, the median (IQR) levels of age, hemoglobin, leukocyte, neutrophil, C‐reactive protein, platelet, prothrombin time, total bilirubin, albumin, alanine transaminase, carcinoembryonic antigen were 60.0 (54.0–66.0) years old, 142.0 (134.0–152.0) g/L, 5.2 (4.3–6.6) * 10^9^/L, 61.2 (54.2–68.9)%, 8.3 (3.4–7.9) mg/L, 148.0 (108.0–188.0) * 10^9^/L, 12.2 (11.7–12.8) s, 13.2 (10.2–16.4) μmol/L, 40.5 (37.7–42.7) g/L, 34.4 (23.5–49.5) IU/L and 236.6 (8.6–1807.4) ng/mL. There were 173 (79.7%) male and 44 (20.3%) female. 154 (71.0%) patients were ECOG score 1 and 63 (29.0%) patients were ECOG score ≥2. Colon cancer patients was 137 (63.1%) and rectal cancer patients was 80 (36.9%).

**TABLE 1:** Baseline characteristics of all patients.

Variables	Number (%)/median (IQR)	Variables	Number (%)/median (IQR)
Sex		PT, seconds	12.2 (11.7–12.8)
Male	173 (79.7%)	TBIL, μmol/L	13.2 (10.2–16.4)
Female	44 (20.3%)	ALB, g/L	40.5 (37.7–42.7)
Age, years	60 (54–66)	ALT, IU/L	34.4 (23.5–49.5)
ECOG score		CEA, ng/ml	236.6 (8.6–1807.4)
0/1	154 (71.0%)	Tumor location	
≥2	63 (29.0%)	colon cancer	137 (63.1%)
HGB, g/L	142.0 (134.0–152.0)	rectal cancer	80 (36.9%)
Leukocyte, * 10^9^/L	5.2 (4.3–6.6)	History of bowel obstruction	
Neutrophil%	61.2 (54.2–68.9)	Yes	38 (17.5%)
C‐reactive protein, mg/L	8.3 (3.4–7.9)	No	179 (82.5%)
PLT, 10^9^/L	148.0 (108.0–188.0)		

Abbreviations: ALB, albumin; ALT, alanine transaminase; CEA, carcinoembryonic antigen; ECOG, Eastern Cooperative Oncology Group; HGB, hemoglobin; PLT, platelet; PT, prothrombin time; TBIL, total bilirubin.

Table 2 shows the basic information of 3 patients with intestinal perforation after receiving bevacizumab. The detailed information of the three patients was summarized as follows:

**TABLE 2:** Baseline characteristics of three patients.

Variable	Case 1	Case 2	Case 3
Sex, male	Female	Female	Female
Age, years	70	59	60
ECOG score	0	0	0
HGB, g/L	104	93	98
Leukocyte, * 10^9^/L	1.20	1.37	1.62
Neutrophil%	82.1	77.6	53.7
C‐reactive protein mg/L	124	5.9	8.3
PLT, 10^9^/L	117	128	136
PT, seconds	11.7	12.6	11.9
TBIL, μmol/L	11.4	18.6	15.8
ALB, g/L	35.8	37.9	36.1
ALT, IU/L	53	26	34
CEA, ng/mL	583.4	15.67	105.9
History of bowel obstruction	Yes	Yes	Yes
Tumor location	right colorectal cancer	rectal cancer	right colorectal cancer
TNM stage	T4N2M1	T4N1M1	T4N2M1
Chemotherapy regimen	mFOLFOX6	FOLFOX4	FOLFOX4 + FOLFIRI
Treatment outcome	died	recovered	died
Dosage of bevacizumab	300 mg	200 mg	300 mg

Abbreviations: ALB, albumin; ALT, alanine transaminase; CEA, carcinoembryonic antigen; ECOG, Eastern Cooperative Oncology Group; HGB, hemoglobin; PLT, platelet; PT, prothrombin time; TBIL, total bilirubin.

### 3.1 Case 1

**FIGURE 1:**
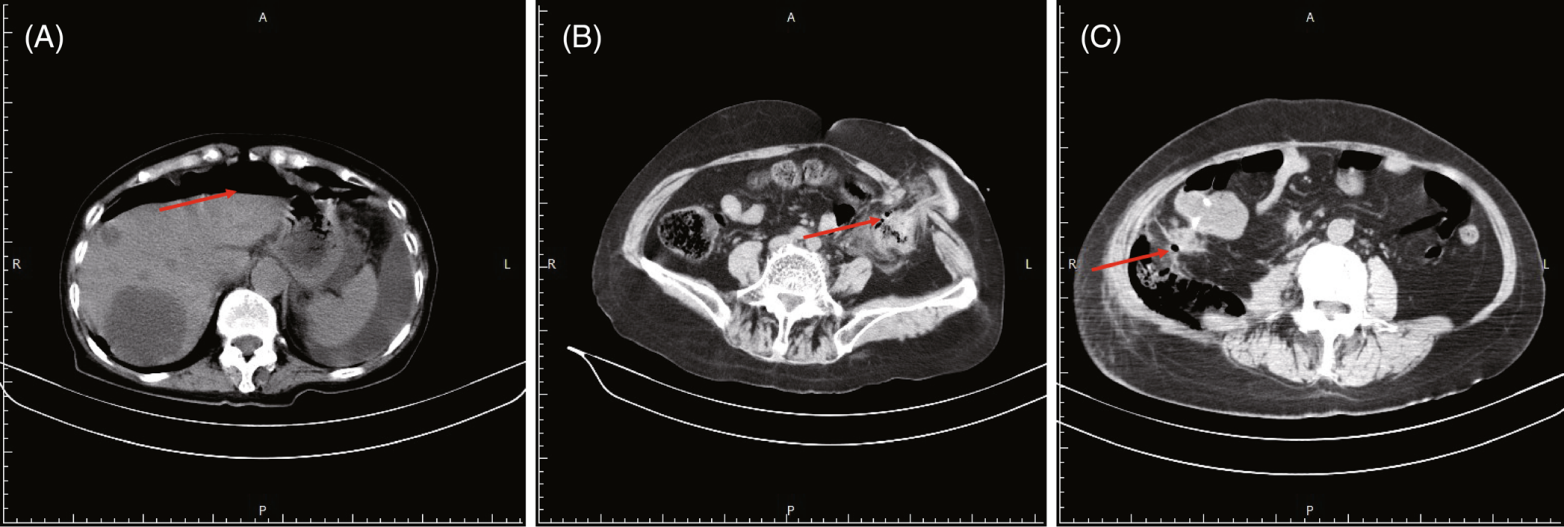
CT of intestinal perforation after receiving bevacizumab. (A) Case 1 images. CT of intestinal perforation after receiving bevacizumab. (B) Case 2 images. CT of intestinal perforation after receiving bevacizumab. (C) Case 3 images. CT of intestinal perforation after receiving bevacizumab.

A 70‐year‐old female was diagnosed with right colorectal cancer. On January 27, 2016, the patient underwent radical resection of colon cancer and liver metastasis. The postoperative pathology showed that colonic tumor and liver metastases were mucinous adenocarcinoma, and colonic tumor invaded peri‐intestinal adipose tissue. Tumor invades microvascular and nerves. The upper and lower surgical margins were negative. There were 7/17 lymph node metastasis in mesenteric root and Para intestinal lymph nodes and two peri‐intestinal cancer node. The pathological stage was pT4N2M1. The patient received 8 course of mFOLFOX6 chemotherapy and bevacizumab (300 mg) after surgery. Then capecitabine was given orally and bevacizumab (300 mg) for 6 courses. On February 28, 2017, the patient received bevacizumab again and then the patient had sudden severe vomiting with persistent middle and upper abdominal pain on March 1. Blood routine showed that C‐reactive protein was 124 mg/L (normal level < 10 mg/L), leukocyte count was 1.20*10^9^/L (normal level 3.5–9.5 * 10^9^/L), the percentage of neutrophil was 82.1% (normal level 40.0%–75.0%), procalcitonin >100.00 ng/mL (normal level<0.05 ng/mL), abdominal computed tomography (CT) was shown in Figure 1A, and the diagnosis was acute intestinal perforation and acute peritonitis. However, considering the advanced tumor stage of the patient, and the general condition is poor. Besides, the chemotherapy drugs and targeted drugs are not fully metabolized, and there are surgical contraindications.^10^ At the same time, the patient's family members did not hope to accept surgery and only give conservative treatment. Finally, the patient's condition progressed and died on March 11.

### 3.2 Case 2

A 59‐year‐old female was diagnosed with rectal cancer. On January 20, 2015, Mile's surgery was performed and the postoperative pathological staging was pT_4_N_1_M_1._ The postoperative adjuvant chemotherapy was given. The patients had perineal pain was present since August 2016, and carcinoembryonic antigen was 15.67 ng/mL (normal level < 10 ng/mL). The pelvic magnetic resonance imaging (MRI) was done and showed that the status after radical resection of rectal cancer and left anterior sacral space occupying lesion with involvement of posterior uterine wall. The patient started receive FOLFOX4 chemotherapy combined with bevacizumab (200 mg) on September 11. Then the patient had left lower abdominal pain, accompanied by fever, nausea and vomit. Subsequently, 150 mL of dark red blood was discharged from the stoma on September 14. The pain persisted for 5 h without significant relief. Then abdominal CT was done and shown in Figure 1B. The preliminary diagnosis was that recurrence of pelvic floor tumor after rectal cancer surgery and intestinal perforation. Then conservative treatment was given and patient recovered successfully finally. After conservative treatment, the patient eventually recovered and was discharged smoothly. The patient continued to receive FOLFOX4 chemotherapy for subsequent treatment, but did not receive bevacizumab. During the chemotherapy period, the patient's tumor was controlled until the chemotherapy was stopped due to bone marrow suppression after 7 course of chemotherapy. Ultimately, the patient's tumor progresses, resulting in multiple abdominal metastases and ultimately death.

### 3.3 Case 3

A 60‐year‐old female was diagnosed with colon cancer. On December 16, 2015, the patient underwent radical surgery for right hemi‐colon cancer. The postoperative pathology showed that tubular adenocarcinoma with grade II‐III. The tumor invades the whole layer of intestinal wall and peri‐intestinal adipose tissue. Cancer thrombus was seen in the lymphatic vessels. The upper and lower surgical margins were negative. There were 8/10 lymph node metastasis in mesenteric root and para intestinal lymph nodes and one peri‐intestinal cancer node. The pathological stage was pT_4_N_2_M_1_. Since January 8, 2016, the patient accepted 10 courses of FOLFOX4 chemotherapy and 6 courses of capecitabine monotherapy orally. On June 30, 2017, positron emission tomography (PET)‐CT was done and showed that the local tumor recurrence in the operation area and multiple tumor metastasis in lung, liver and spleen, multiple tumor metastasis in abdominal cavity and right pleural tumor invasion. Then the patient underwent 1 course of FOLFIRI chemotherapy and bevacizumab (300 mg) on July 3, 2017. On July 22, 2017, the patient had sudden swelling and pain in the right waist, accompanied by nausea and retching. The skin of the right waist was red and swollen, with a range of 10 * 10 cm and accompanied by obvious tenderness. The blood routine showed that the leukocyte count was 1.62 * 10^9^/L (normal level 3.5–9.5 * 10^9^/L), the percentage of neutrophil was 53.7% (normal level 40.0%–75.0%), platelet count was 136 * 10^9^/L (normal level 125–350 * 10^9^/L), and abdominal CT was showed in Figure 1C. The preliminary diagnosis was that postoperative colorectal cancer, multiple tumor metastasis and intestinal perforation. Considering the advanced tumor stage of the patient and the general condition was poor. At the same time, the patient's family members did not hope to accept surgery and only give conservative treatment. Finally, the patient's condition progressed and died on July 24, 2017.

## 4 DISCUSSION

Colorectal cancer is a common gastrointestinal malignancy, accounting for the first incidence rate of gastrointestinal tumors.^1^ Approximately 35% of patients with colorectal cancer present with distant metastases at the time of diagnosis, and the prognosis of these patients is poor.^14^ Chemotherapy combined with targeted therapy is currently the common clinical treatment for metastatic colorectal cancer. Of the several types of targeted agents now used for colorectal cancer patients, only bevacizumab has been associated with bowel perforation. Bevacizumab is a targeted drug that can specifically bind and block epidermal growth factor receptor (VEGF), and plays a role through the mechanism of inhibiting angiogenesis. VEGF is the key driver of tumor angiogenesis, which is the basic process of angiogenesis and maintenance. And it is necessary for tumor growth and metastasis to other parts of the body. The precise target effect of bevacizumab can help control tumor growth and metastasis without aggravating the adverse reaction of chemotherapy. Therefore, the combination of bevacizumab with chemotherapy for the treatment of metastatic colorectal cancer can maximize the clinical effect and improve the survival of patients.^15^ However, the use of bevacizumab can lead to adverse effects, such as hypertension, fatigue, diarrhea and so on.^16^ The incidence rate of gastrointestinal perforation was low, but it is one of the most serious complications, which may lead to higher mortality. The incidence of gastrointestinal perforation after receiving bevacizumab was reported to be 0%–2%.^9^ Kabbinavar et al. reported the use of bevacizumab in 1953 patients with metastatic colorectal cancer. The result showed that 37 patients (1.9%) occurred gastrointestinal perforation.^11^

In this study, there were 217 patients with metastatic colorectal cancer received bevacizumab. The result showed that 3 patients occurred intestinal perforation, with a crude incidence rate of 1.4%. The specific mechanism and risk factors for intestinal perforation after receiving bevacizumab are unclear. It has been suggested that the possible mechanisms leading to gastrointestinal perforation as follows. The first possible mechanism was that bevacizumab has inhibitory effect on VEGF, which can also induce the production of coagulation factor III, von willebrand factor and plasminogen activator inhibitor. After treatment with bevacizumab, the above‐mentioned coagulation factors were inhibited, which could lead abnormal coagulation mechanism.^17^ The abnormal coagulation mechanism will lead to the embolism of visceral microvascular or mesenteric vessels, which will lead to intestinal ischemia and necrosis, and finally lead to the occurrence of gastrointestinal perforation.^18^ The second possible mechanism was that after the tumor cells of colorectal cancer invade the intestinal mucosa, it will cause the tumor cells to adhere to the intestinal wall.^17^ The use of bevacizumab could affect the stability of the intestinal wall and eventually leading to gastrointestinal perforation.^19^ The last possible mechanism was that bevacizumab could inhibit the proliferation and healing of intestinal wall cells, and lead to gastrointestinal perforation eventually.^18^

**FIGURE 2:**
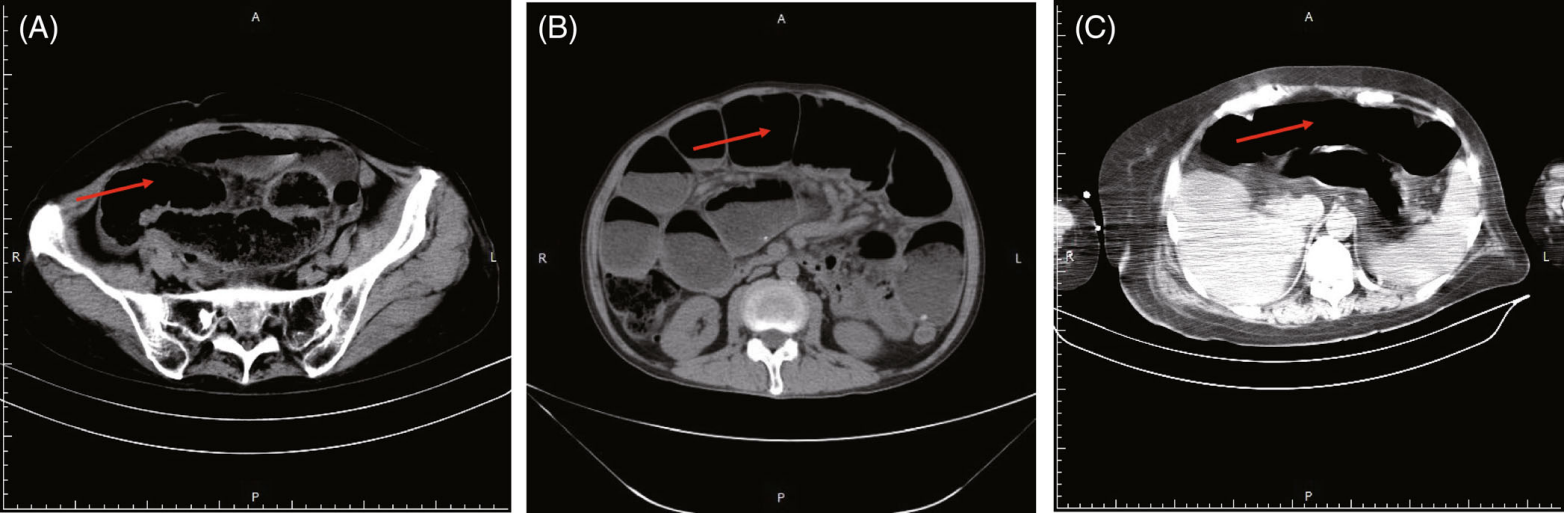
CT of intestinal obstruction prior to perforation. (A) Case 1 images. CT of intestinal obstruction prior to perforation. (B) Case 2 images. CT of intestinal obstruction prior to perforation. (C) Case 3 images. CT of intestinal obstruction prior to perforation.

The risk factors for gastrointestinal perforation after receiving bevacizumab are currently considered to be related to age, the dose of bevacizumab and the patient's past medical history. Kabbinavar et al. reported that patients younger than 65 years have high risk of gastrointestinal perforation when receiving bevacizumab.^11^ In our study, 2 patients were younger than 65 years old and 1 patient was older than 65 years old. All three patients who occurred intestinal perforation were female patients, suggesting that female patients may have high risk of intestinal perforation than male patients. In addition, there may be a correlation between the occurrence of intestinal perforation and the patient's history of intestinal obstruction. The three patients with intestinal perforation reported here all had varying degrees of intestinal obstruction prior to perforation (Figure 2A,B,C), suggesting that patients with intestinal obstruction prior to the application of bevacizumab may have high risk for intestinal perforation. Of course, the specific mechanisms and risk factors for intestinal perforation with bevacizumab are not fully understood and further research is needed.

Surgery and conservative treatment are commonly used for patients with intestinal perforation after receiving bevacizumab. However, patients with advanced colorectal cancer are generally in poor condition due to the advanced stage of the tumor. If these patients undergo surgery, the risk of surgery is high. Therefore, doctors need to be more cautious when choosing surgeries for these patients.^20^ Badgwell et al. reported that 19 of the 24 patients with gastrointestinal perforation after receiving bevacizumab accepted conservative treatment and three patients eventually died within 30 days.^20^ In our study, three patients of intestinal perforation all accepted conservative treatment and then two patients died and one patient recovered.

## 5 CONCLUSION

Patients with advanced colorectal cancer after receiving bevacizumab are at risk of gastrointestinal perforation. Although the incidence rate is low, once it occurs, the prognosis is very poor. The patient's age, gender and history of bowel obstruction may be associated with intestinal perforation after receiving bevacizumab. Therefore, it is necessary to further investigate the risk factors associated with gastrointestinal perforation after receiving bevacizumab and to adequately assess the risk of gastrointestinal perforation in patients after receiving bevacizumab. The bevacizumab should be used with caution for patients with high risk of gastrointestinal perforation. And follow‐up and monitoring during administration should be strengthened to detect and treat patients with gastrointestinal perforation caused by bevacizumab in time.

## AUTHOR CONTRIBUTIONS

Guarantor of the article: Minmin Sun, Yong Xia.

Kunpeng Fang, Jie Wang, Jianyong Yuan, Chengjun Sui, Jiajun Zhi, Yong Xia and Minmin Sun contributed in study concept, data collection, and drafting of the manuscript; Kunpeng Fang, Jie Wang and Jianyong Yuan contributed in data search and extraction; Minmin Sun, Yong Xia contributed in study concept, design, drafting of the manuscript and study supervision.

All authors approved the final version of the manuscript.

## FUNDING INFORMATION

This study was funded in full by the Program of Science and Technology Commission of Shanghai Municipality (grant number 21Y11912700).

## CONFLICT OF INTEREST STATEMENT

The authors declare no conflicts of interest.

## ETHICS STATEMENT

The treatment plan was according to clinical guidelines.

## CONSENT TO PARTICIPATE

All patients obtained informed consent before treatment. And the written informed consent of each patient was obtained for publishing their clinical results.

## CONSENT FOR PUBLICATION

Not applicable.

## Data Availability

Data sharing is not applicable to this article as no new data were created or analyzed in this study.
